# The sex- and duration-dependent effects of intermittent fasting on lifespan and reproduction of spider mite *Tetranychus urticae*

**DOI:** 10.1186/s12983-019-0310-4

**Published:** 2019-04-11

**Authors:** Guang-Yun Li, Zhi-Qiang Zhang

**Affiliations:** 1 School of Biological Sciences, University of Auckland, Auckland, 1072 New Zealand; 2 Landcare Research, 231 Morrin Road, Auckland, 1072 New Zealand

**Keywords:** Fasting, Sex-specific, Longevity, Reproductive efforts, Trade-off

## Abstract

**Background:**

Intermittent fasting (IF) is receiving increasing attention as an alternative to continuous restriction of calories because of its benefits in aging-related disease prevention and lifespan extension. However, whether both sexes with sexual dimorphism have similar response to IF have rarely been assayed. In this study, we determined how different durations of IF influence lifespan and whether males and females differed in their responses to IF. We also tested whether there is a trade-off between lifespan and lifetime reproduction in females under IF.

**Method:**

We used spider mite *Tetranychus urticae,* with female-biased sexual size dimorphism (SSD), as our model species to investigate the survival and lifespan difference of both sexes at different durations of IF regimes, and explore the association between longevity and fecundity in females within and across treatments.

**Results:**

The lifespan of females increased before intermediate level of IF and then decreased afterwards, but males showed a decreasing trend in lifespan when subjected to IF. Within each treatment, female longevity was positively associated with their fecundity. However, the females fed *ad libitum* had a higher lifetime fecundity with a shorter lifespan, whereas mites fed 50% IF outlived *ad libitum* fed ones with lower fecundity because of the later onset of reproduction and lower daily fecundity, showing clear survival and reproduction trade-off when variation of resource availability enhanced across treatments.

**Conclusion:**

We showed sex-specific response to IF in lifespan, indicating that sexes with SSD have different optimal level of IF. These findings showed trade-off between survival and reproduction between treatments but not within treatments, suggesting that variation in resource availability is the necessary precondition for life history trade-off, and IF extends lifespan of females at the cost of reproductive success.

## Background

Aging is a biological process modulated by both genetic architecture passed down from parents and environmental factors the animal experiences throughout their lives [1–3]. To date, a wide array of anti-aging interventions has been proposed to extend healthy lifespan and enhance the health status, e.g. dietary restriction (DR), exercise, pharmacological interventions, and hormesis [4, 5]. Among these, dietary restriction, defined as reducing food intake without malnutrition, is the first and most reproducible intervention supported by a large body of empirical studies in the last few decades [6–8]. The concept of DR has been expanded from initial calorie restriction to a wide range of diet-related intervention including short-term starvation, intermittent fasting, and macronutrient (i.e. necessary amino acid, proteins, carbohydrates) restriction [6].

Intermittent fasting (IF) has drawn great attention recently and was proposed as an alternative to traditional dietary restriction [6, 8–10]. During intermittent fasting, animals are periodically exposed to fasting during which the food is withdrawn. IF differs from DR in that it does not limit the amount of food available. Instead, the animals are offered food ad libitum at scheduled feeding periods [10, 11]. DR and IF are both reported to be beneficial for model organisms and for humans—the most direct and obvious one being the promotion of weight loss [12, 13]. In addition, they also generate many physiological benefits, including enhancing glucose tolerance [14], decreasing heart rate and blood pressure [15, 16], and reducing oxidative damage [10], which results in the extension of a healthy lifespan and a lower incidence of aging-associated diseases such as cardiovascular disease and kidney disease [10, 17–19]. DR seems intuitively more challenging than IF to implement for experimental animals; the latter is thus gaining increasing popularity and requires further investigation as a potential protocol for delaying aging and enhancing healthy lifespan.

While it is quite obvious that dietary interventions have several health benefits for animals, their effects on lifespan demonstrated in previous studies are not always consistent [20]. In some long-lived animals such as mice, IF (alternative day fasting) beginning at the age of 1–2 months increased adult lifespan [21]. Similar results were also observed for short-lived rotifers with an average lifespan of 2 weeks [22]. However, some previous studies on IF showed contradicting results. A well-controlled investigation with fruit fly (*Drosophila melanogaster*) showed no positive effects of IF [23]. But a most recent paper showed that IF can benefit fruit fly with longevity extension [24]. Additionally, in one study with short-lived fish, IF even significantly shortened the longevity in comparison with ad libitum fed controls [25]. This disparity in the effects of IF on lifespan for different taxa might be due to their differences in reproductive modes, which have rarely been addressed in most studies.

Although the effects of these dietary interventions on aging and lifespan have been explored extensively across a diverse range of animal taxa, ranging from invertebrates (e.g., *Drosophila melanogaster* and *Caenorhabditis elegans*) to vertebrates (e.g., *Rattus rattus, Mus musculus*, and *Macaca mulatta*) [26], the underlying mechanism by which dietary restriction modulates aging process has yet to be understood [2]. Given that an increased longevity in response to dietary restriction has been witnessed to be coupled with a decrease in fecundity in laboratory animals, this phenomenon appears to indicate that a trade-off between reproduction and survival is the driving force of longer lifespan, which played a crucial role in theory and interpretation of the life history studies [27]. The most prominent theory is the disposable soma theory, which suggests that these two fitness components share a finite resource pool, thus an increased investment in reproduction would withdraw resources that might otherwise be available for somatic maintenance [28–30]. Because previous research on aging mainly focused on longevity, few experimental studies have investigated the influence of dietary restriction on reproduction. As a result, more empirical support for the trade-off theory is still needed. Moreover, the limited existing evidence is mainly demonstrated in model organisms including fruit fly, worm, and mice, which showed a more remarkable response to dietary restriction than non-model organisms [31]. Therefore, further investigations that explore the impact of the dietary restriction on both longevity and reproduction are needed, particularly in other non-model species.

It has recently been noticed that males and females are not only different in aging rates and lifespan within species, but also display distinct responses to a variety of anti-aging interventions [32, 33]. In many studies, DR has weaker effects on males than on females, both in the model and non-model species [31]. Moreover, there is a sex-specific optimal food regime for lifespan extension. For example, in the *D. melanogaster,* the females have a longer lifespan on average than the males, and their longevity reached a plateau at a food concentration of 60% of the standard laboratory diet compared with a concentration of 40% for males [34]. One possible reason is that males generally invest less in reproduction, and they consequently need comparatively little resource for somatic maintenance and reproduction. However, there is relatively little information about how dietary regimes influencing lifespan of males differently from females.

In this study, we investigated the influence of IF on lifespan and reproduction in a non-model species: a spider mite (*Tetranychus urticae*),which belongs to the Acari (mite) family Tetranychidae It exhibits cosmopolitanism and has a wide range of host species, including beans, peppers, tomatoes, potatoes, corn, cannabis, and strawberries, with beans being one of its preferred host plants. At 25 °C, the spider mites usually develop from egg to adult in about ten days, and the adults live for around two weeks with lifetime fecundity ranging from 50 eggs to 178 eggs [35]. Although there are extensive documents on the life history traits of female spider mites, the male has been largely ignored. We included both males and females as subjects, aiming to examine the sex difference in aging by exposing them to three treatments of IF with controls fed ad libitum. Our previous study found that bigger females have higher starvation resistance than small males [36]. Therefore, we expected that the effects of IF would be level and sex dependent, and the longevity and reproduction of males and females would differ across the treatments. Furthermore, we predicted that the long-lived female would show a lower lifetime fecundity as predicted by the life history trade-off model. Specifically, there would be a negative correlation between female longevity and lifetime reproduction.

## Material and methods

### Stock culture

The mites used in this study are two-spotted spider mites *T. urticae,* a plant-feeding animal. The stock culture was derived from a small population obtained from Bioforce Ltd., New Zealand in February 2015. It was maintained under well-controlled conditions in a greenhouse room at Landcare Research, Auckland, New Zealand. This population was reared on potted common bean plants (*Phaseolus vulgaris L.*), a favourable host plant. Newly potted bean plants (about 4 weeks old) were regularly added to the stock culture.

The potted bean plants were sowed every week and cultured in a separate insect-free room in the greenhouse to make sure there were enough fresh bean plants for the mite population. Both the mite population and plants were kept at 20 ± 5 °C, 60 ± 10% RH and the natural photoperiod in all seasons except for winters, with a cycle of 16 h light:8 h dark.

### Experiment protocol

In our previous starvation tolerance test, *T. urticae* males and females experienced a higher risk of mortality after 2 and 4 days of food deprivation, respectively [37]. According to this result, we deduced that 2 days fasting is the maximum both females and males can tolerate without extremely deleterious effects. Therefore, the spider mites were exposed to three treatments of IF, i.e. 33% IF, 50% IF, and 67% IF by feeding them 2 days out of every 3 consecutive days (feed-feed-starved; abbreviated as 33% IF hereafter), 2 days out of every 4 days (feed-feed-starved-starved, 50% IF), and 1 day every 3 days (feed-starved-starved, 67% IF), with spider mites feed at ad libitum as control. They were transferred to a rearing arena with a black plastic sheet as a substrate during starvation, while placed on fresh leaf discs as a substrate during the feeding period. Although feeding one day out of every two days (feed-starved-feed-starved) was an alternative for 50% IF (2 days out of every 4 days i.e. feed-feed-starved-starved), we chose the latter because the difference in man-made interference during transferring mites among IF treatments could be minimized this way (each mite must be moved 3 times in feed-starved-feed-starved but only once in feed-feed-starved-starved).

The experiment started with males and females on the first day of the final molt. To prepare a large population of mites at this age, the unmated females and mated females from the lab population were transferred to leaf discs to lay eggs for 24 h, and these eggs of the same age were then collected and allowed to hatch and develop into adults of similar ages. In all treatments, males and females were paired and fed during the first 2 days of adulthood. They underwent the first starvation period on the third day after the adult emergency when most females started to produce eggs. The mites were then exposed to the four dietary regimes: ad libitum, 33% IF, 50% IF, 67% IF, as mentioned above. For all treatments, the leaf discs were changed every 4 days to make sure there was fresh food for mites. The experiment was conducted with 24 well cell culture plates with one pair of mites (male and female) in each cell. The sample size ranged from 23 to 44 for each treatment. During the experiment, two indices including survival and female reproduction were measured. The survival of each mite was checked every 24 h until all mites were dead. The number of eggs produced by each female was recorded each day. The experiment was conducted at 25 ± 2 °C, 65 ± 10% RH and a photoperiod cycle of 16 h light:8 h dark.

#### Statistics

All statistical analyses were conducted in R software version 3.4.2. For the IF experiment, the survival data were first fitted in the Cox proportional-hazards model with treatment (4 levels) and sex (2 levels) as independent variates. Since the interaction between sex and treatments was significant (*P* < 0.05), the survivorship was compared between treatments, for females and males respectively. Pairwise comparison was conducted using Kaplan-Meier method with log-rank test, taking the censored into consideration. R packages ‘survival’ and ‘survminer’ were employed for computing survival analyses and visualizing the results, respectively. The adult longevity was normally distributed, so two-way ANOVA test (R function ‘aov’) was applied to check the main effects of sex, treatment, and the interaction between them.

The female reproductive parameters, including pre-oviposition period (time period from adult emergence of a female to its first egg being laid), oviposition period (the time period from the first egg of a female being laid to the last egg being laid), post-oviposition period (the time period from the last egg of a female being laid to its death), daily reproductive rates (the number of eggs produced by female per day), lifetime fecundity (total number of eggs produced by a female), maximum daily reproductive rate (the largest number of eggs produced by a female during its life), and the female age at maximum daily reproductive rate (age of the female when a female showed maximum daily reproductive rate), were compared with dietary regimes as the main factor using R function ‘aov’. Post-hoc comparison was performed with TukeyHSD. To clarify the relationship between adult longevity and lifetime fecundity, analysis of covariance (ANCOVA) was conducted with longevity as dependent variable, fecundity as independent variable, and IF tratments as covariates (R function ‘aov’). As there was significant interaction between longevity and IF treatments, linear regression (R function ‘lm’) was performed for each treatment, respectively.

## Results

### Effects of IF on the survival of males and females

**Fig. 1:**
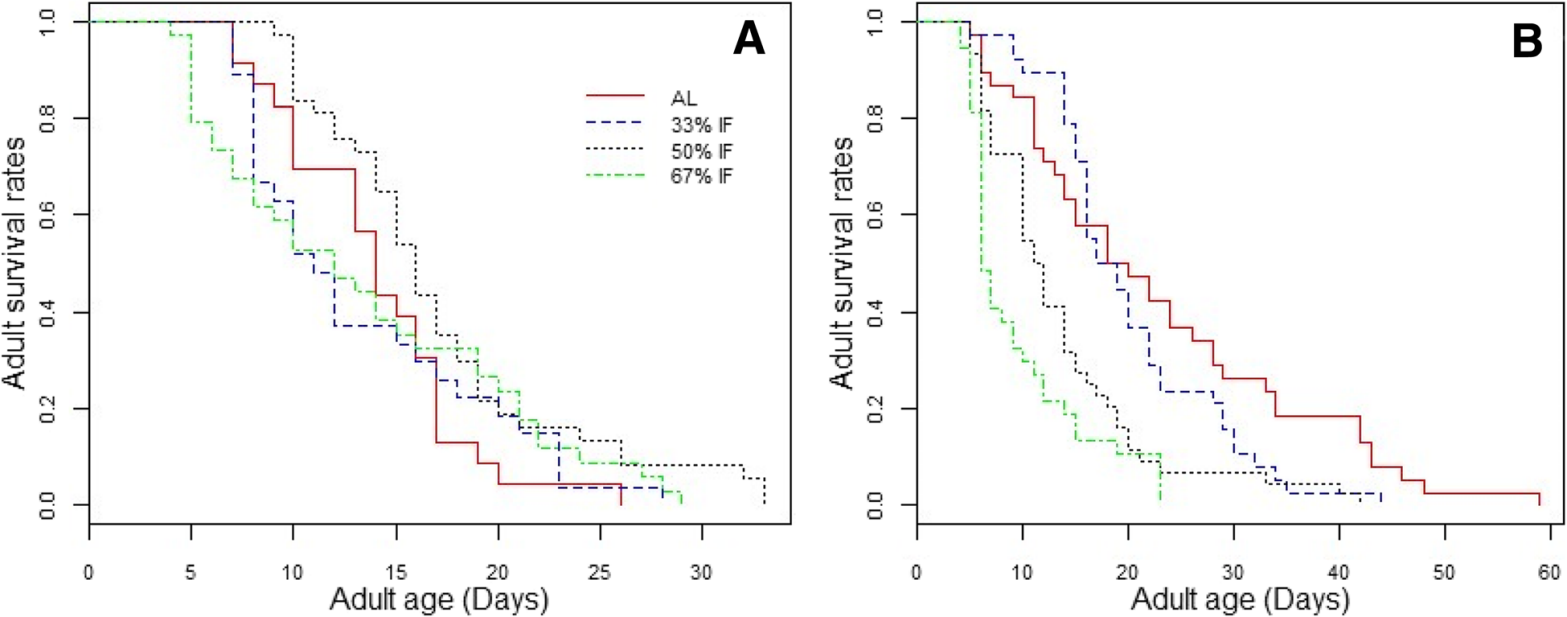
Survival curves of spider mites *T. urticae* under a different treatments of intermittent fasting (IF). **a** Survivorship of female *T. urticae* subjected to three treatmentsof IF in comparison with that of mites fed ad libitum. N_*AL*_ = 23, N_33%IF_ = 27, N_50%IF_ = 34, N_67%IF_ = 37. **b** Survivorship of male *T. urticae* subjected to three treatments of IF in comparison with that of mites fed ad libitum. N_*AL*_ = 38, N_33%IF_ = 38, N_50%IF_ = 44, N_67%IF_ = 37. Panel **a** and **b** share the same legend

The spider mite demonstrated sex-specific response to IF, with females having a higher survival rate at modest fasting, while males having a reduced survival rate under all levels of fasting. The survival rate of female spider mites fed at these four dietary regimes showed significant difference, with the females fed at 50% IF showed highest survival, followed by mites fed ad libitum, and mites on fasting for 1 day or 2 days every 3 days (χ^2^ = 9.1, *df* = 3, *P* = 0.03, Fig. 1a). Specifically, the ad libitum fed females had a similar survival to those fed at 33% IF and 67% IF (χ^2^ = 0.3, *df* = 1, *P* = 0.6 and χ^2^ = 0.1, *df* = 1, *P* = 0. 8, respectively), but a marginal difference compared with females fed at 50% IF (χ^2^ = 4.3, *df* = 1, *P* = 0.04). These results suggested that the optimal regime for lifespan extension of female spider mites was around 50% IF, and neither a lower nor a higher level of fasting was effective in extending lifespan.

In contrast, for the males, the survivorship decreased with the increasing durationsof fasting. Males exposed to 33% IF had a similar survival compared with those fed at ad libitum (χ^2^ = 3.3, *df* = 1, *P* = 0.07, Fig. 1b), but males fed at 50% IF and 67% IF had significantly lower survival rates than the control males (χ^2^ = 20.5, *df* = 1, *P* < 0.001 and χ^2^ = 33.6, *df* = 1, *P* < 0.001), indicating that males were very sensitive to fasting and showed a negative response to the severe fasting.

### Effects of IF on the life span of males and females

**Fig. 2:**
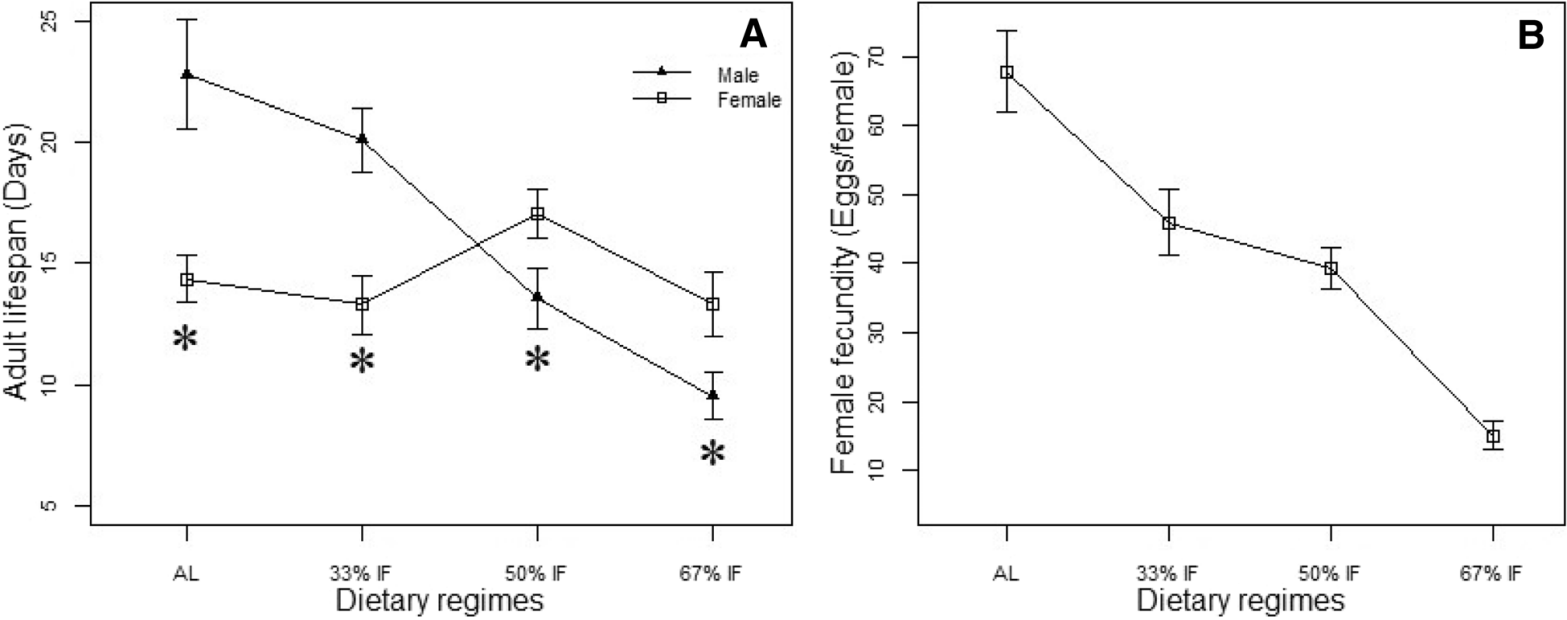
Mean lifespan and lifetime fecundity of spider mites *T. urticae* under a different treatments of intermittent fasting (IF). **a** Effects of feeding regimes: ad libitum, 33% IF, 50% IF, and 67% IF on the adult life span of female and male spider mites. Data were shown in means±se (days). The asterisk denote there was significant difference in lifespan between male and female at each level of food regime. For the female: N_*AL*_ = 23, N_33%IF_ = 27, N_50%IF_ = 34, N_67%IF_ = 37. For the male: N_*AL*_ = 38, N_33%IF_ = 38, N_50%IF_ = 44, N_67%IF_ = 37. **b** Effects of feeding regimes: ad libitum, 33% IF, 50% IF, and 67% IF on female reproductive rates of female spider mites N_*AL*_ = 23, N_33%IF_ = 27, N_50%IF_ = 34, N_67%IF_ = 37.. Data were shown in means ± SE (eggs/female). Panel **a** and **b** share the same legend

Both IF and sex showed significant influence on the lifespan of spider mites. With the increase of IF durations, the adult longevity of spider mites (with male and female pooled together) decreased from 18.41 ± 1.10 days to 11.42 ± 0.99 days (*F*_3, 270_ = 8.352, *P* < 0.001, Fig. 2a). The males showed a reduction in longevity with IF level elevated, while female longevity peaked at 50% and then decreased (Fig. 2a). Moreover, dietary regimes had a significant interaction with sex on adult lifespan (*F*_3, 270_ = 10.612, *P* < 0.001). The males fed at ad libitum and at 33% IF lived significant longer than the corresponding females (*AL: t* = 3.58*, df* = 48.68*, P* = 0.001 and 33%IF: *t* = 3.82*, df* = 62.74*, P* < 0.001), whereas at higher level of IF, 50% IF and 67% IF, the females significantly outlived the males (50%IF: *t* = 2.14*, df* = 78.18*, P* = 0.03 and 67% IF: *t* = 2.33*, df* = 61.76*, P* = 0.02; Fig. 2a).

### Effects of IF on reproductive parameters

There were no statistical differences in the durations of the pre-oviposition period and oviposition period, even though the mites on IF seemed to delay the onset of the reproduction (Table 1). In addition, no significant variation was shown between female mites fed on these four different food regimes on post-oviposition periods (Table 1).

**Table 1:** Effects of dietary regimes on reproductive period and investment of female spider mites

Parameters	Ad libitum	33% IF^1^	50% IF	67% IF	*P*
Pre-oviposition period (days)	1.13 ± 0.07a^2^	1.41 ± 0.14a	1.65 ± 0.14a	1.63 ± 0.19a	*P = 0.113*
Oviposition period (days)	12.21 ± 0.86a	11.80 ± 1.19a	14.57 ± 0.97a	11.28 ± 1.29a	*P = 0.067*
Post-oviposition period (days)	2.13 ± 0.44a	1.00 ± 0.00a	2.06 ± 0.28a	1.52 ± 0.16a	*P = 0.081*
Daily reproductive rate (Eggs/day)	5.60 ± 0.31a	3.75 ± 0.13 b	2.77 ± 0.10c	1.33 ± 0.55d	*P < 0.000*
Maximum reproductive rate (Eggs/day)	9.52 ± 0.46a	8.53 ± 0.32b	7.62 ± 0.28c	2.94 ± 0.12d	*P < 0.000*
Adult age at MRR^3^ (days)	5.41 ± 0.66b	7.47 ± 0.61ab	8.85 ± 0.68a	5.90 ± 0.60b	*P < 0.000*

Note: ^1^The abbreviations 33% IF, 50% IF, 67% IF denote feed for 2 consecutive days at 3-day interval, 2 consecutive days at 4-day interval, 1 day at 3-day intervals

^2^Means (±SE) within the same row followed by the different letters are significantly different (*P* < 0.05)

^3^MRR is the abbreviation for the maximum reproductive rate

N_*AL*_ = 23, N_33%IF_ = 27, N_50%IF_ = 34, N_67%IF_ = 37

**Fig. 3:**
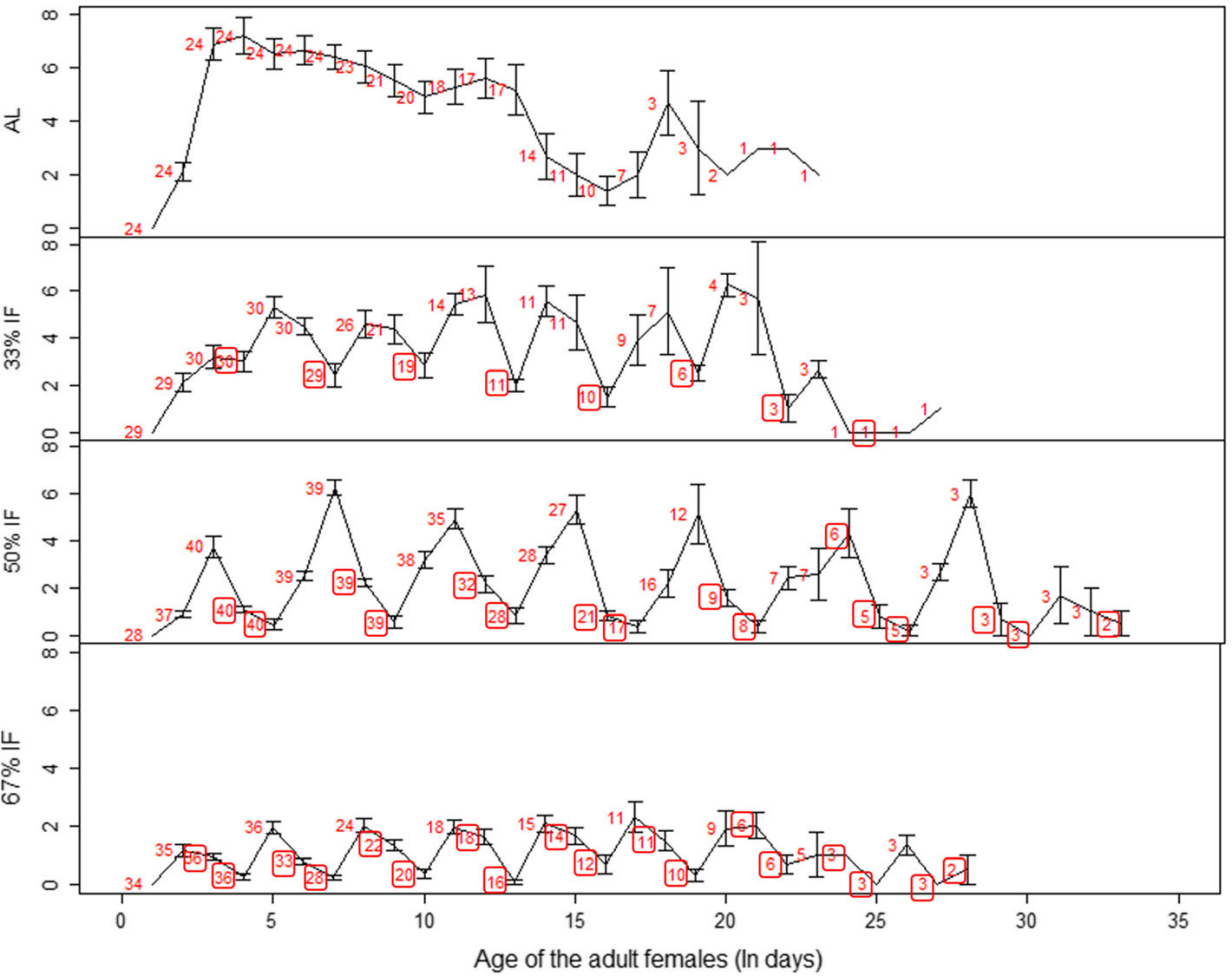
The average daily reproductive rates (Mean ± SE) of adult females feeding on the four different dietary regimes: ad libitum, 33% IF, 50% IF, and 67% IF. The red numbers beside the line denote the sample size of each data point, and these numbers within boxes indicated the number of mites on starvation. The sample size increased in the earlier stage with more and more females starting to lay eggs, but in the later stage it decreased with the death of some females

Although food regimes did not have a significant influence on female reproductive lifespan, the fecundity showed a remarkable response to it by decreasing monotonically with the increasing level of IF (Fig. 2b). The daily reproductive rate, estimated as the mean number of eggs produced by a female per day, decreased significantly with increasing level of food deprivation, so did lifetime fecundity (Table 1). On average, the females fed ad libitum produced four times as many eggs as the mites exposed to 67% IF. The maximum daily reproductive rate was the highest for females fed ad libitum, followed by 33% IF, 50% IF; it was the lowest when mites experienced 67% IF. The females’ maximum daily reproductive rate was witnessed at the age of 7.47 days and 8.85 days when fed at 33% IF and 50% IF, respectively, much older than those fed ad libitum and 67% IF. The daily reproductive rate distribution curve further indicates that when females were deprived of foods for 2 consecutive days, their eggs were mostly produced during the feeding period. During the fasting period, egg production decreased on the first day and even ceased on the second day of fasting (Fig. 3).

**Fig. 4:**
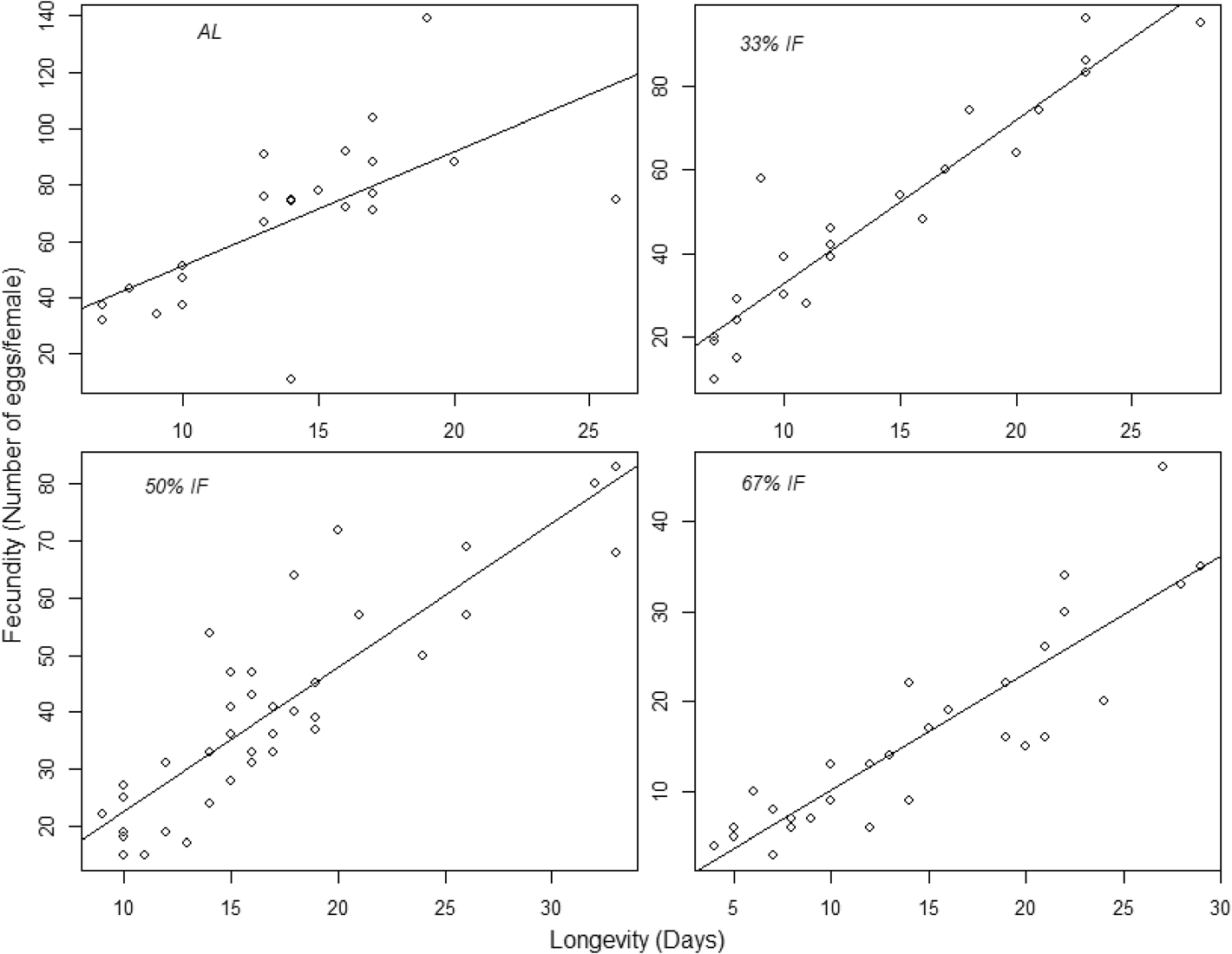
Correlations between female adult life span and lifetime fecundity for spider mites fed at four different feeding regimes. Note that axis scales are different for each treatment. N_*AL*_ = 23, N_33%IF_ = 27, N_50%IF_ = 34, N_67%IF_ = 37

The ANCOVA analysis indicated that both dietary regimes (*F*_3,106_ = 69.91, *P* < 0.001) and longevity (*F*_1,106_ = 130.73, *P* < 0.001) have significant effects on the lifetime fecundity, and their interaction is significant (*F*_3,106_ = 11.97, *P* < 0.001). Correlation analysis was used to assess the correlation of lifetime fecundity and adult lifespan of females showed significant positive relationships when the females are pooled together regardless of food regimes (*F*_1,112_ = 45.94, R^2^ = 0.2845, *P* < 0.001). When food regimes were taken into consideration, and the correlation were checked across the four feeding regimes, there was also a significant positive correlation between these two parameters for each treatment (ad libitum (*F*_1,21_ = 15.75, R^2^ = 0.4014, *P* = 0.001), 33% IF (*F*_1,22_ = 175.6, R^2^ = 0.883, *P* < 0.001), 50% IF (*F*_1,35_ = 108.9, R^2^ = 0.749, *P* < 0.001), and 67% IF (*F*_1,28_ = 107.3, R^2^ = 0.785, *P* < 0.001), Fig. 4).

## Discussion

With the emerging evidence indicating that dietary restriction is an efficient protocol extending healthy lifespan, more and more dietary regimens are being explored because the daily food restriction is difficult to implement in practice [18, 37, 38]. In this study, we examined the effects of IF, an alternative to DR, on the fitness of spider mites. The male and female spider mites experienced IF of different fasting durations showed distinct responses. Specifically, the females fed at a moderate level of IF (50% IF) lived longer than the ad libitum fed spider mites with a lower life time reproduction and older age at maximum reproductive rate, while males showed a decrease in lifespan with increasing durations of IF. Although between treatments the longer lifespan is associated with lower fecundity and the delayed onset of maximum reproductive potential, the fecundity showed a positive correlation with the lifespan in female spider mites within each treatment.

### Sex difference in lifespan and response to IF

The males and females demonstrated a sex-specific response to different treatments of IF. With the increased level of fasting, females first showed extended lifespan at 50% IF than the controls (ad libitum feeding), but as fasting regimes (67% IF) levelled, their lifespan shortened. However, the fasting males exhibited shorter lifespan compared with the control and lifespan were further shortened with increasing level of fasting. This is the first report demonstrating a sex difference in response to IF, as the majority of existing research investigating the effects of IF have ignored the potential sex-specific response.

One possible explanation for the sex-specific response to IF is their differences in nutrient reserve and resource allocation. On the one hand, the females are able to store much more nutrients and energy, and thus compensate for the food shortage better during fasting periods. This is supported by the fact that females are more resilient to starvation when food is deprived without further food replenishment, as shown in our previous study and some other research [35, 39, 40]. On the other hand, from the daily observation of female fecundity, it was clearly shown that when there was no access to food, females could reduce reproduction on the first day and even stop reproduction on the following fasting day. This may indicate that the females can divert the limited resource from reproduction to somatic maintenance [41]. In contrast, not only do the males not have as much nutrient reverse as the females, but their reproductive performance is also less likely to be influenced by diet. This assumption can be further tested by measuring the triacylglyceride (TAG) levels across different treatments and sexes. In a recent study, it has been reported that TAG levels decreased in fruit fly (*Drosophila melanogaster*) subjected to 5-day IF [24]. Given the importance of body nutrient reserve in animals under IF and the lack of information on data about it, it might be interesting to investigate whether there is a sex difference in nutrient reserves such as lipid storage.

Another potential factor is their difference in gene expression when experiencing dietary intervention. Although the female and male are almost similar in genetic architecture, they vary in gene expression, which can possibly generate sex-specific difference [42]. This has already been tested in *Caenorhabditis elegans,* which is a model organism that displayed lifespan extension in response to DR. Under dietary intervention, the hermaphrodites and males differ in expression levels of gene encoding key enzymes involved in carbohydrate metabolism. The males have around twice as many sex-specific gene expression than do the hermaphrodites, and 35% of all C-type lectin genes have higher expression levels in males [43]. Understanding the physiological difference associated with gene expression variation between sexes maybe crucial for understanding the mechanism of sex-specific aging. Additionally, another study with *C. elegans* indicated that the longevity advantage of hermaphrodites over males was influenced by the terminal transcription factor (TRA-1) of the nematode sex-determination pathway, which can activate the expression of daf-16/ FOXO to regulate development and lifespan [44]. However, whether the protein TRA-1 is evolutionary conserved and functions in other species warrants further investigation.

### Duration-dependent response to IF

In some empirical studies on dietary intervention, the animals are only treated with one level of IF with controls fed ad libitum [e.g. 25]. The protocols of IF applied are usually borrowed from other studies without being tailored to the species involved in a new study. This might be one of the explanations why there is a disparity in existing evidence. For example, every other day feeding regime can be effective in extending lifespan of some rotifers like *Brachionus plicatilis* [22]*,* but it was reported to be less effective or even detrimental to other species of rotifers such as *Cephalodella spp.* and *E. worallii* [41]. Moreover, fruit fly *Drosophila melanogaster* experienced starvation for 3 h and 6 h per day failed to show longevity extension [23]. In contrast, fruit fly females benefited with longer lifespan when starved for 5 days per week in early adult stage. The males, which was more starvation sensitive, also significantly increased lifespan when fasted for 4 consecutive days per week [24]. These studies suggested that the effects of intermittent fasting was duration and sex dependent. Therefore, as discussed above, a well-designed fasting regime considering factors such as sex and their difference in starvation tolerance is of great importance in further exploring the influence of IF on health and lifespan.

### Relationship between lifespan and reproduction

Most previous research on intermittent fast only concerns survival and lifespan, few attempted to explore its potential influence on reproduction. In this study, we assayed the reproductive success of spider mites at different food regimes and the relationship between longevity and reproduction within and between treatments. On the one hand, within each population, a positive linear correlation was found between longevity and lifetime fecundity. The low quality females died early with fewer number of offspring whereas the high quality females lived longer and produced more eggs. Consequently, the high individual heterogeneity in resource acquisition masked the entailed cost of reproduction. On the other hand, across treatments, the females under IF showed a significant decrease in their fecundity with increasing duration of fasting, which is consistent with findings on rotifer *E. worallii* [41]. But among all the investigated levels of food regime, the lifespan of females reached a peak at 50% IF, and decreased when fasting levelled up and leading to malnutrition. Clearly, the females reduced daily fecundity and even ceased reproduction during food deprivation. This indicated that under moderate resource limitation (50% IF) the spider mite diverted the energy and nutrient from reproduction to self-maintenance, in line with the leading hypothesis that longevity extension under fasting is an adaptive plastic response of animals reallocating resource from reproduction to somatic. Furthermore, the long-lived females at 50% IF also demonstrated an older age at maximum reproductive rate, suggesting food restriction retarded the pace of reproduction in comparison with the shorter-lived counterparts fed ad libitum. Collectively, despite the individual heterogeneity masked trade-off between survival and reproduction within each population, clear trade-off was demonstrated among treatments, whereby the spider mites fed ad libitum with abundant resource showed highest lifetime reproduction with a shorter lifespan while females at 50% IF with limited resource prolonged lifespan at the cost of reproductive success.

### Spider mite as potential candidate for ageing research

With a series of empirical studies using spider mites, including this one, we underlines the suitability of *T. urticae* as a model species in gerontology and anti-ageing research. First, it has the common characteristics as the classical model organisms such as nematode and fruit fly in that it develops fast, can be easily to be reared in the laboratory and has low cost for population maintenance [45]. It is also an excellent candidate for genetic investigation because its genome has already been sequenced and annotated, making it a promising model organism for the genetic research on aging. Its small genome distributed on three holocentric chromosomes is 75 Mbp [46], only 60% of *Drosophila* genome and 80% of *C. elegans* genome. In addition, the well-established RNAi-mediated gene silencing technology for spider mite by injection of dsRNA [47] makes it possible to generate loss-of function phenotypes and investigate the function of specific genes.

## Conclusions

In this study, we extended previous work in the field of IF by taking the sex of subjects and durations of fasting into consideration, representing the first experimental evidence that the response of lifespan to IF is sex and duration dependent. First, the study highlights the fact that females and males are of different sensitivity to starvation and fasting; accordingly, they differed in their patterns of aging when exposed to a series of IF regimes. Other animals with sexual dimorphism may also have a sex-specific response to IF. Second, the reproduction of females decreased across all IF treatments, but lifespan extension was demonstrated in females at intermediate fasting level 50% IF. The females at 50% IF with longer lifespan showed decreased lifetime reproductive success and older age at MRR, which suggests there are substantial costs in reproductive fitness for life span extension in females. One of the common challenges for studies in this field is that although costs of life span extension are well documented in females, few observations have been reported in males, possibly owing to the protocol challenge of measuring reproductive efforts of males. Further work revealing the differences in the relationship between lifespan and reproduction between sexes may give insights into the underlying mechanism of sex-specific response to diet intervention. Additionally, whether the influence of intermittent fasting can be carried over to the next generation, affecting the quality of eggs and their hatching rates as well as other fitness traits of offspring, warrants further investigation.

## Abbreviations

DR: dietary restriction, defined as reducing food intake without malnutrition, is the first and most reproducible intervention supported by a large body of empirical studies in the last few decades. The concept of DR has been expanded from initial calorie restriction to a wide range of diet-related intervention including short-term starvation, intermittent fasting, and macronutrient (i.e. necessary amino acid, proteins, carbohydrates) restriction.
IF: Intermittent fasting, is a feeding pattern that cycles between a period of fasting and non-fasting period.
MRR: Maximum reproductive rate is the highest daily fecundity of a female in throughout life.
SSD: Sexual size dimorphism, is the difference of body size between male and female of the same species.
